# The Meaning of Musicing in the Post-traumatic Growth of Individuals With Adventitious Visual Impairment: Applying the Life History Method by Mandelbaum

**DOI:** 10.3389/fpsyg.2021.690771

**Published:** 2021-07-08

**Authors:** Hye Young Park

**Affiliations:** Music Therapy Department at Kosin University, Busan, South Korea

**Keywords:** adventitious visual impairment, musicing, post-traumatic growth (PTG), life history, Mandelbaum

## Abstract

This study investigated individuals with adventitious visual impairment (AVI) acquired during adulthood through a traumatic event, for an in-depth and contextual understanding of the factors and processes that led to positive changes in their post-traumatic growth. The life history method was applied on 15 individuals with AVI (seven males and eight females) through in-depth interviews about their life. The study’s analytical framework involved three domains: dimensions, turnings, and adaptations of life, as proposed by Mandelbaum. The results revealed the following key factors: of the dimensions of life—family, rehabilitation center, and music groups; of turnings of life—positive change through awareness and tolerance of impairments, new challenge posed by rehabilitation training, and finding inner resources through music; and of adaptations of life—accepting one’s life in a harsh social environment, actively establishing relationships with others as an individual with visual impairment, and re-finding meaning of life through musicing. This study contributes by revealing the role of each of the above-mentioned factors and identifying their correlations. The results suggest that musicing may help individuals with AVI develop empathy and engage in social communication through active self-disclosure.

## Introduction

Vision is an important sense that enables humans to acquire information about objects and the surrounding environment, not only to determine the quantity but also obtain certainty about the information. Thus, individuals with visual impairment may experience limited ability to acquire information as well as physical threat, psychological anxiety, feelings of helplessness and isolation, and a severed relationship with the outside world. Visual impairments pose a challenge to individuals in terms of social behaviors, the learning of which mostly requires observation and imitation; and discomfort in communicating or empathizing with individuals who have normal vision, and this may limit their chance to participate in the society or form interpersonal relationships (Codding, 2000; Tuttle and Tuttle, 2004; Sacks and Rosenblum, 2006; Alma et al., 2012). Carroll (1961), a renowned educator of individuals with visual impairment in the United States, said, “Loss of sight is dying,” thus defining visual impairment as a severe form of loss. This view implies that a physical disability may take away the life of an individual and degrade the meaning of his/her entire life, by exerting a negative influence on his/her emotional, psychological, and social behaviors. Despite this negative definition of visual impairment, some people with visual impairment actively use other senses, especially auditory sense, to compensate for their lack of vision; this allows them to enjoy their lives to the same degree as those with normal vision (Park, 2017). As an auditory medium, music can play an important role in the life of people with visual impairment such as providing enjoyment, social communication, and empathy with others (Robb, 2003; Rostohar, 2006). Most people with visual impairment are more comfortable and dependent on their auditory sense (Molloy-Daugherty, 2013), and music as an auditory stimulus could be utilized to expand their psychological resources and establish positivity in their lives. Although there have been many studies conducted focusing on music and visual impairment, the meaning and role of music in the lives of the visually impaired have been rarely identified.

### Adventitious Visual Impairment

Individuals with adventitious visual impairment (AVI) are those who have acquired their visual impairment in midlife, although the definition may be generalized to include those who acquired visual impairment during adulthood (Bajaj, 2019). The psychological trauma caused by loss of vision is felt to a greater extent by individuals with AVI than those with congenital visual impairments (Yang and Park, 2016; Haegele and Zhu, 2019; Jaleel et al., 2019). The former experience events that seem to deny their life and even their very existence, as they see their daily life destroyed, lose their job, and undergo various problems in their relationships with family and colleagues. As they face the challenges that markedly differ from the type of challenges they faced before the visual impairment, individuals with AVI are required to newly adapt to not only the external factors, including personal physical changes, but also internal factors, including personal values in life (Jaleel et al., 2019).

### Post-traumatic Growth

Post-traumatic growth (PTG) refers to the positive psychological changes that are subjectively felt by the trauma-affected individual (Calhoun and Tedeschi, 1999). An individual who experiences PTG goes on to find new meanings and possibilities in life, different from the time before the traumatic event. This enables them to interpret life with new perspectives while overcoming the associated pain, as they place greater values on their new relationships and recognize their inner power with increased interest on spiritual aspects (Tedeschi and Calhoun, 1996). Common factors in the experience of growth are personal inner power, interpersonal relationships, new possibilities in life, gratitude toward life, and spirituality (Tedeschi and Calhoun, 2004). Hence, PTG comprises a set of positive changes that an individual experiences as a result of his/her efforts to overcome the trauma he/she experienced. For individuals with AVI, the loss of vision during their midlife could be regarded as a traumatic event, and such individuals can experience PTG while overcoming the pain and hardships brought about by the loss of vision. In fact, previous studies regarding individuals with visual impairment report the severe psychological trauma caused by loss of vision and the post-traumatic stress reaction to the traumatic event (Brunes et al., 2018; Kim and Yoo, 2018; van der Ham et al., 2021).

### Musicing

Music is a field of art that enables individuals with visual impairment to apply other senses actively, such as the auditory sense. It represents a field that can be enjoyed by individuals with visual impairment to a level identical to individuals with normal vision (Tuttle and Tuttle, 2004). In fact, a considerable number of individuals with visual impairment have been reported to use music not only for personal enjoyment but also as an effective means of empathy with others (Molloy-Daugherty, 2013) and of social communication (Wolffe and Sacks, 1997; Robb, 2003). In other words, as music can be enjoyed largely through the auditory sense and thus, gives wide and varied aesthetic experiences, it may help individuals with visual impairment to form emotional interaction with others (Rostohar, 2006).

Meanwhile, Elliott (1995) described the essence of music as the act and process of an individual creating music rather than the music itself. Thus, music comprises the musicer, the musicing, and the music as the product; and the true nature of music is not simply about the product with a specific value but every event in the course of the detailed creation by the musicer. Hence, music’s true meaning lies not in the object represented by the music as the product but in the creative activity of *musicing*, for which the process, that is, the musical experience, rather than the product is the most valuable part. *Musicing* is an active way in which human beings relate to the rest of the world through direct experience of expressive and receptive processes, and it acts an interactive gesture with which they can communicate, respond to others, learn, participate in, and explore themselves (Small, 1999).

For individuals with visual impairment who depend on their auditory sense as an alternative, complementary sense is high, and for those whose auditory sense has thus been enhanced to a substantial degree through efforts to overcome the visual impairment, music or *musicing* may provide a sense of autonomy through independent activity, satisfaction through accomplishment, and social communication or empathy, thereby having a positive influence on their life (Lee, 2015). Notably, as individuals with AVI strive to overcome severe psychological and emotional challenges, the creative, aesthetic, expressive, and transcendental power of music as a complementary experience may represent a psychological resource, or provide the energy to support their life (Aigen, 2005).

### Purpose of the Study

The present study conducts an in-depth analysis of how PTG is experienced by individuals with AVI through *musicing* from an insider’s perspective. Compared with individuals with congenital visual impairments, individuals with AVI face the shock and pain of a sudden loss of vision in midlife, before which they had no disability. Meanwhile, the feelings of loss, depression, and frustration they experience could be steered toward new meanings and possibilities in life through *musicing*. Therefore, this study investigated the factors and processes of PTG that led the participants (individuals with AVI) of this study to experience positive changes in life after the event that caused their loss of vision, with a particular focus on the meaning of *musicing* throughout their PTG. An in-depth understanding of how individuals with AVI experience PTG through *musicing* will provide basic data for developing practical interventions to help these individuals live on as healthy members of the society.

For this study, among the life history methods, the analytical framework of Mandelbaum was applied, as it involves the analysis of multidimensional domains of life beyond a simple chronological analysis (Mandelbaum, 1973). Thus, the following research questions were addressed:

First, what *dimensions of life* of individuals with AVI are maintained through musicing?

Second, what *turnings of life* of individuals with AVI are related to musicing?

Third, what strategies do individuals with AVI implement by means of *adaptations of life* relating to musicing?

## Method

In this study, the PTG of individuals with AVI was analyzed using the life history method. The study aimed at an in-depth and contextual understanding of the growth factors that lead to personal and social environmental changes, specifically, the positive psychological changes throughout an individual’s life. In contrast to other qualitative studies where the analysis focuses on a specific point of time in an individual’s life, the life history method is characterized by an analysis that simultaneously takes into account temporality, the changes that occur, and continuity. The method thus allows an analysis of the stories throughout an individual’s life, with the benefits of accurate data on a participant’s course of life and high reliability of the collected data (Giudice et al., 2015).

### Participants and Data Collection

For ethical considerations regarding the participants, the potential subjects were given adequate explanations of the study purpose as well as the participant’s rights and the privacy policy, and those who signed written consent for voluntary participation were selected. The participants of this study were individuals with visual impairment (AVI, level 1–3 only) totaling 15 adults (seven males and eight females) aged ≥ 18 years and with AVI caused by an accident or disease. To ensure anonymity of the participants, pseudonyms were used, and their general characteristics are presented in Table 1.

**TABLE 1:** General characteristics of study participants (*N* = 15).

Name (pseudonym)	Gender	Age (in years)	Cause of AVI	Age at VI (in years)	Occupation
Ghi-Ho	M	52	Diabetic retinopathy	40	Massager
Hye-Soo	F	33	Retinitis pigmentosa	26	Unemployed
Do-Hoon	M	55	Behcet’s disease	35	Manager
Mi-Rhim	F	43	Glaucoma	30	Teacher
Myeong-Gyu	M	38	Optic atrophy	25	Social worker
Nam-Geon	M	45	Retinal detachment	32	Massager
Ah-Rhan	F	39	Retinitis pigmentosa	32	Lecturer
Yeon-Jin	F	40	Macular degeneration	31	Massager
Dong-Geun	M	33	Retinal detachment	26	Social worker
Da-Hee	F	40	Glaucoma	29	Massager
Jong-Goo	M	42	Retinal detachment	34	Office worker
Yoon-Ji	F	36	Behcet’s disease	29	Barista
Min-Seon	F	46	Macular degeneration	40	Massager
Ji-Seon	F	47	Diabetic retinopathy	32	Homemaker
Jeong-Ho	M	58	Glaucoma	43	Social worker

*AVI, adventitious visual impairment; VI, visual impairment.*

The subjects were recruited from the X Community Welfare Center, which has 30 years of experience in rehabilitation training of individuals with AVI. The center has provided various rehabilitation programs for the participants such as Braille-learning, orientation and mobility, activities for daily living training, sports, culture and leisure programs including singing, playing instruments, writing poems, dancing, and vocational rehabilitation. Based on the trainers’ recommendations, 26 individuals with AVI who satisfied the study criteria (i.e., who are currently participating in an activity related to music) were selected. The researcher contacted each of the 26 individuals—whose contact details were provided by the trainers after checking their willingness to receive a call or be visited upon request—to explain the study purpose and ethical policies. Fifteen individuals with AVI (adults aged from 33 to 58 years) who agreed to participate were finally selected. The criteria used are as follows.

• Level of disability: restricted to levels 1–3 of visual impairment

(Levels of visual impairment were classified according to the Ministry of Health and Welfare of Korea’s criteria. Level 1: best-corrected visual acuity ≤ 0.02; level 2: best-corrected visual acuity ≤ 0.04; level 3: best-corrected visual acuity ≤ 0.08.)

• Age at onset: adventitious

• Experience with music: currently participating in musical activity more than 2 h a week

The data collection was performed for approximately four months from July to October in 2020. The interviews were mostly performed at locations designated by the participant such as cafés, work places, practice rooms, and bible study rooms, as they may feel inconvenienced if asked to move from place to place. For each individual, two interview sessions were conducted, each lasting 90 min on average, and a complementary interview was performed upon request or when necessary. In some cases, phone interviews were performed, consisting of three among thirty (2 sessions per 15 participants) 90 min sessions upon request and eight short phone calls for additional questions. The recorded interviews as digital audio were transcribed into text format by the researcher.

### Data Analysis

The data collected through the interviews were analyzed based on the analytical framework of three aspects proposed by Mandelbaum (1973), which includes *dimensions of life*, *turnings of life*, and *adaptations of life*. Mandelbaum claimed that the life history method should move on from the level of descriptive analysis to a more in-depth level of analysis. Such a critical view led Mandelbaum to propose a method of analysis based on the basic framework of three concepts: *dimensions*, *turnings*, and *adaptations*, thus complementing the previous descriptive method that simply lists the life history in chronological order (Park, 2017). Mandelbaum stated that for the life history method to provide more useful data, the multiple dimensions of life should be analyzed in addition to a simple chronological analysis.

For this, interviews were conducted with an unstructured questionnaire in which the participants talked about their life process and life experiences using their own voices. The unstructured interview is a valuable method that helps identify themes and concepts in a broad and in-depth manner (Schreiber and Stern, 2001). This helps the researcher understand the life histories of participants in the appropriate context (Lee, 2011). The interview questions used in this study were “How was your life before and after the onset of visual impairment?”, “What does visual impairment mean to you?”, and “How does musical activity work in your life?”.

The analytic process for the collected data utilized three stages: data reduction, data display, and derivation/confirmation of conclusions. Data reduction refers to the processes of selecting, focusing, simplifying, summarizing, and transforming transcript data. Data display includes information organization and compression to derive conclusions and provide the researcher with methods of understanding what is happening; the results are typically displayed based on the various formats available. With regard to the derivation and confirmation of conclusions, the researcher determines the meanings of objects and events and focuses on the analysis of patterns, regularities, explanations, possible forms, and causal flows. These three stages can be regarded as description–analysis–interpretation, proposed by Wolcott (1994). In the text transcripts of the interviews, the researcher found meaningful concepts and key events, and repeated text reconstruction until the organic relationships between the themes were revealed (Silverman, 2016). Finally, the researcher extracted common concepts that were consistently found in the life history of 15 participants of this study.

First, for the *dimensions of life*, three factors (family, rehabilitation center, and music groups) with a direct influence on the participants’ PTG were analyzed. Second, for the *turnings of life*, details related to the key factors in the experience of positive changes through awareness and tolerance of visual impairment and the new challenges posed by rehabilitation training were analyzed. Third, for the *adaptations of life*, the source of energy that drives individuals with AVI to lead life with such strong will against all odds was identified, while an in-depth analysis was conducted on the meaning of musicing that ultimately led the participants to achieve PTG.

### Strictness of the Study

To ensure reliability of the study results, peer debriefing and member checking were performed (Stemler, 2001). First, in the peer debriefing designed to maintain objectivity, as a pilot test, samples were drawn to measure the inter-rater reliability. It is suggested that 10% or 20% of the entire set be used as samples to test the inter-rater reliability (Wimmer and Dominick, 1997), while 5–7% is also regarded suitable (Kaid and Wadsworth, 1989). Thus, in this study, 20% was applied for the test of reliability, which was performed by a PhD researcher in the field of music therapy and who has an experience in conducting a qualitative study. For the items of disagreement in the first inter-rater reliability test, the raters and the researcher had a thorough discussion, after which the reliability in the second test was 92.5%. Next, in the member-checking phase, the aim was to re-evaluate the validity of the study results, and for this, the analysis results and conclusions drawn from the study by the researcher were sent via email to eight study participants, who were requested to give their opinions and evaluate the validity of the results. Through this process, the participants were able to examine the results directly, and this enhanced the accuracy of the study contents and analysis.

### Ethical Considerations

To observe the research ethics guidelines in a manner that protects the research participants’ privacy and their possible confrontation of negative memories, the researcher has applied conversation, integrity, and connection of the criteria as proposed by Sikes (2010). The researcher continually initiated conversations through person-to-person sessions and partner-like relationships, maintained the integrity of life stories by collecting qualitative data and in-depth interviews, and finally tried to relate these stories by linking trajectories of AVI inner resources, positivity, and new relationships with the given socio-cultural context. Considering the psychological and emotional difficulties that the participant may experience by recalling traumatic events during in-depth interviews, the researcher performed all interview sessions after initiating prompt interventions from a mental health expert.

## Results

Nine common concepts were derived, as these were consistently shown by the 15 individuals with AVI, and they were detected through the life history method.

### Analysis of the Dimensions of Life

In the life history method, the dimensions of life represent more than just simple livelihood; they reveal how an individual has survived up to the present moment. For the participants of this study, the dimensions of life that enabled them to live through the various relationships and changing social environment and culture from birth to the present were found to be their family, rehabilitation center, and music groups. As the most crucial resources that enabled these individuals to endure through and live up to the present moment, these three dimensions formed the core of their existence, and at the same time, served as the fundamental driving force for their ceaseless efforts and fighting spirit to respond to future challenges.

#### Family

For the participants of this study, the family was their most reliable support group and the refuge they could always go back to for comfort. The family was also the source of positive energy that motivated individuals with AVI to face the harsh reality and endure through hardships, as they suddenly acquired visual impairment in midlife and received rehabilitation training. Their family was the pillar they could lean on, despite constant feelings of guilt and apology. As in other people’s lives, their family was not particularly special to them before the impairment, but afterward, their family became the most important dimension of their life, protecting and supporting them. The family was the source of energy these individuals needed to continue on with the challenge, and to grow despite insecurities about the future.

“I used to work in a heavy equipment rental business when I lost my vision. I received rehabilitation training at X Community Welfare Center, and my wife visited me once a week during that time. She always brought the sashimi that I like the most in a cooler for me. Now I am running a massage parlor with several employees who are visually impaired like me. My wife manages all the laundry such as bed sheets, pillow covers, and towels, because I can’t see if they are too old or have holes since I lost my vision. I can’t imagine living another day without my wife. I am truly grateful, and feel sorry for her, always.” (Ghi-Ho).

“I didn’t study hard at high school and frankly, I didn’t like it. So I couldn’t go to college. I worked as a sales person at a department store, and during my sixth year at that job, I became visually impaired. I am unable to work now, so I live with my parents, and I think my mum cries a lot. She seems to be full of worries because I don’t have a job. I feel so sorry and ashamed, but living with my parents has given me a great deal of energy and I really get a lot of help from them.” (Hye-Soo).

#### Rehabilitation Center

For the participants of this study, the rehabilitation center was another dimension in their life that played a central role in their daily activities from the moment their vision got impaired. It presented possibilities for these individuals whose vision was suddenly impaired, with the center providing practical support for them to continue to lead an active life despite feeling frustrated about being losers, and for them to interact with other people in the society. For these individuals who had to face unforeseen changes due to their visual impairment, the rehabilitation center was a place where they could find those facing the same challenges and with whom they can interact, to share the pain and provide consolation and encouragements; this helped them face the reality of their impairment and plan their future with hope.

“I was working at a major company when my visual impairment happened. A colleague of mine introduced me to X Community Welfare Center, and on the day I visited the center with my wife, I felt that the staff were really kind. I used to feel alone, suddenly becoming a person with impaired vision, but at the center, I saw that there were many people just like me. It was great relief for me that I could talk to those facing the same problems I face, about things that I couldn’t tell anyone else. I still often call them. When we get together, we chat a lot and drink sometimes.” (Do-Hoon).

“When I was studying abroad I became visually impaired. Soon after I came back to Korea, a friend of my brother introduced me to X Community Welfare Center. I visited the center and entered their education program. I learned Braille and how to use screen reader programs for the visually impaired there. Last year, they helped me get a cane for women that is much thinner and lighter than I used before; I am using it now and I love it. Even after completing my education, they still reach out to me and care about me. I feel truly grateful to them.” (Mi-Rhim).

#### Music Groups

The music groups in which all the 15 participants of this study are an active member (choir, vocal band, orchestra, church choir, etc.) served as a shelter away from their weary life, a place of comfort where they could momentarily forget about the frustrating and sometimes sad constraints of their daily life, and a constant source of positivity that invigorated them. In meeting those with the same disability or those without impairment and singing and playing instruments together, these individuals with AVI seemed to broaden their horizons and gain energy to overcome the fear they frequently felt. They created music with the members of the music group to which they belonged, thereby finding reasons to live on, discovering hope, and drawing energy to face the extraordinary reality of their impairment.

“After about six months of playing a wind instrument in a military band, I lost my vision. I work at the Community Welfare Center now, and whenever I feel weary and stressed, I think about Sunday afternoon [band] practices, and I always smile. There are seven of us in the band, four with visual impairment, including me, and three with normal vision who are college friends of mine; we practice in the basement of the Center, and it is great fun. At first, we weren’t very good, but we’ve now been together for a year and a half, we played a song by The Beatles last month. Our hope is to have a concert of our own next year.” (Myeong-Gyu).

“When I was working at the department store, I often went to Karaoke with my friends. I was the best in our group. Now in our choir, I sing the soprano part with a friend of mine with visual impairment, and people tell me I have a beautiful voice. Living with my parents, I often feel sorry, ashamed, and guilty, but here, with the choir, I can forget about all those negative feelings. I am amazed that I can do this well, and people’s compliments are a huge support.” (Hye-Soo).

“I lost my vision in an accident while working at a construction site. It was at the Community Welfare Center where I learned to play the drums through their music education program. When I am absorbed in playing, I can feel my anger and stress dissipating. Even though my playing is still clumsy, I am the loudest instrument in our band. The others tell me to be quiet when we practice, and it is all so enjoyable and I feel happy. I hope to be in the band for a long time.” (Nam-Geon).

“I learned to play a string instrument when I was young, and these days, I play in a local amateur orchestra with school teachers. It is a bit difficult to memorize the score, but everyone is considerate as I am the only one with visual impairment. At the end of the rehearsal, when we play a piece from the beginning to the end, I feel a sense of accomplishment and happiness for completing the piece, which I suppose is only possible through music.” (Mi-Rhim).

### Analysis of Turnings of Life

The analysis of the turnings of life targets the entire life of each participant, as proposed by Mandelbaum (1973). The turnings—that had a direct influence on the life before and after, their trigger, meaning, significance, relation to present life and influence on the different choices taken in the face of reality—were examined. The life of an individual was viewed as a cycle based on the close correlations among events rather than a simple, linear flow of time. Individuals with AVI were found to have lived a life of an ordinary person or achieved economic success that draws everyone’s envy, in spite of suddenly acquiring visual impairment. Considering the time these individuals spent in overcoming the related challenges and the fact that they must live every day feeling the same burden, accepting and overcoming the reality of impairment was found to be the key factor for them. This was beyond personal experience, choice, or decision, but rather, in direct or indirect interaction with different conditions, including their relation to the society, the social conventions within a specific culture in South Korea, and social prejudices toward individuals with AVI or the disabled in general. In the context of life, to accept and overcome the impairment was the central theme throughout the life of these individuals, from the very beginning up to the present time.

#### Positive Change Through Awareness and Tolerance of One’s Impairment

The participants of this study experienced positive changes through awareness and tolerance of their impairment. To be precisely aware of, be tolerant of the impairment, and change their thoughts and actions for their future life is a matter of identifying for themselves what they would continuously face throughout their life. For individuals with AVI, the question is not about what they want to do but about what they can do, having suddenly acquired visual impairment and being subject to limited conditions in their social environment, culture, values, and social conventions. An active attitude to achieve awareness and tolerance of their impairment and positive changes in their life are the true turnings of life, as well as real-life tasks that they continuously face.

“I studied vocal music at university, married my husband who also majored in music, and in my early 30s, I became visually impaired. After that terrible event, my husband disappeared and now I don’t know where he is. It was—or rather, is—a tragedy in my life. I am currently working at the Welfare Center for the Blind in X city, and every day for me is like going to war. Going to and from work is really challenging, but I muster up the courage, thinking that I should be thankful I have a job and people who need me, and when I come home to have dinner, I say I had a good day, I should keep on, and one day, something good might happen to me.” (Ah-Rhan).

“Graduating from a college, I worked at a small shop for more than 10 years before my visual impairment. Now I work at a massage parlor. When I began work, my hands and arms were painful, but these days I am accustomed to it. My clients say that they feel rejuvenated after I massage them; the elderly love my service because I can locate the precise spot that causes them pain. I feel like I’m living a new life, so I feel happy at times and say to myself that my life is really quite good, that I should do my best in this life, accepting that the visual impairment has already happened.” (Yeon-Jin).

“I went on a winter hike to celebrate my graduation from university, and I fell from a cliff. I was in the hospital for more than half a year, but in the end, I lost my vision. Now I work at a local branch of the organization for the visually impaired in X city. My work is rewarding, as it involves helping those with the same visual impairment. Last year, our branch was given five cars by X city to support the mobility of visually impaired people in the community. I wrote that proposal, so I was very pleased. I am hoping for a better tomorrow, while staying calm to resolve daily tasks and challenges.” (Dong-Geun).

#### New Challenge in Rehabilitation Training

For those who used to have normal vision and rely on their vision to do everything, a sudden loss of vision denies them their previous life, causes fear of the future, and poses new challenges, and those holding on to a positive mindset choose to receive rehabilitation training. For the study participants, the training provided them with techniques for life after rehabilitation as they continuously overcame challenges, while they retained hope and encouraged themselves, thinking they will get used to this one day. For individuals with AVI who had to change the habits, lifestyle, thoughts, and even occupation they had before the impairment, the rehabilitation training was the turning of life that gave them hope with regard to the functional aspect of PTG.

“I worked at a big company, and I became visually impaired in my late 20s. An acquaintance introduced me to X Community Welfare Center, where I received rehabilitation training. In the center I learned how to walk alone with a cane and how to use software for the visually impaired. It is still quite inconvenient for me to walk around with a cane on my own, but I have improved greatly compared with how it was at the beginning. I work at a massage parlor now, and without rehabilitation training, I would probably be sitting at home, sighing away my time and feeling helpless.” (Da-Hee).

“When I lost my vision I thought I wouldn’t be able to do anything. I even thought, ‘Can a blind person eat by himself?’ After rehabilitation training at X Community Welfare Center, I am now able to walk around alone with a cane, although clumsily, and I can use a smart phone, too. My accomplishments are tremendous. Now I can call anyone and also send messages anytime I want. I am getting better and better every day.” (Jong-Goo).

“I studied cooking in college, ran a restaurant with two of my friends that my parents had supported money for opening, but in my late 20s, I lost my vision. I received training at a rehabilitation center, where I learned everything from braille, gait, and how to use software for individuals with visual impairment. I acquired a barista certificate two years ago, and now I work at a cafe on the first floor of the Welfare Center for the blind in X city. I am truly happy when people drink the various beverages I serve and hear them say that the aroma is good or the flavor is special. I want to run my own cafe one day, so I am working hard and saving money. With hope in my heart, the work is always fun.” (Yoon-Ji).

#### Finding Inner Resources Through Music

For the participants of this study, music was an aesthetic tool that enabled them to find their inner-resources and use these resources to achieve a positive lifestyle. Music was a source of happiness that invigorated them to discover a dream to become someone and hope to do something, and be a significant member of the society, where the true value of one’s self could be shared in healthy and robust ways. The process of finding one’s inner resources—the potentials they had, the new possibilities they defined, and the uniqueness that became apparent only after the visual impairment—through music was a true turning of life that taught these individuals to keep their chin up and never feel hopeless in heading toward the future.

“I was in a band in high school and I played the keyboard. I worked at a big company until I lost my vision at my early 40s. Several other individuals with visual impairment and I formed a band last year, and it is wonderful as I am the leader. Even though more than twenty years have passed, my experience in high school band still gives me confidence to lead my group. I feel like I’m living a new life, leading this band.” (Min-Seon).

“My father bought me a guitar as a birthday gift when I was in the first year of middle school. I had forgotten about it for a long time; going to college, the army, running a business, and then, in rehabilitation training at X Community Welfare Center, I picked it up again. Now, I play for the choir at church with several players, and I feel happy. I made a lot of church friends and completely stopped drinking, and perhaps in spiritual terms, I feel I am renewed.” (Ghi-Ho).

“We have a choir at our branch where I sing the bass part; there are three men and four women, a total of seven members. We frequently perform at local events. Last year, we sang for an event at a care home, and the elderly loved it so much that they invited us to come back the following year. They thanked us, and I felt true happiness in singing and making people happy, regardless of my visual impairment.” (Dong-Geun).

### Analysis of Adaptations of Life

Adaptation is a part of life, which, in a progressive sense, comprises continuous changes. The most frequently mentioned words for the PTG of individuals with AVI were positive, relationship, value, and meaning. The process in which individuals who have suddenly acquired AVI accept the reality of life in a harsh social environment, to lead relationships with others and to rediscover the values of life through musicing; all these signify a proactive attitude toward a different dimension beyond the adaptation to a new life. To overcome the trauma of visual impairment and accept it in a positive light to achieve growth, to establish relationships with others and use the aesthetic resource of musicing to rediscover the values of life, are creative adaptation strategies that enable finding significance in the given environment, culture, and conventions, and allow assuming social roles.

#### Accepting My Life in a Harsh Social Environment

The most important adaptation strategy chosen by individuals with AVI in this study was “accepting” or “accommodating.” They were aware of the harsh environment that surrounded them, and through experience, they understood that such challenges could not be readily resolved. However, they also understood that—to accept and overcome the impairment and to take a positive approach in their current life (despite its vast difference from the past) with hope for the future—taking initiative in creating their reality rather than just waiting and seeing the social environment overpower it was the only way to achieve growth both on the personal and social levels.

“My husband and sons, and especially my parents, worry about me greatly. When I lost my vision, I could not accept it at all. But I think it’s amazing how well I get on with life and I take pride in that. Although my husband and two of my sons help me a lot, I do all the cooking, not to mention all the household chores. I am the wife and mother in this house; that has remained the same, before and after visual impairment. I am slowly getting better and getting used to it. It is my life, and I should live it sincerely, and enjoy it thoroughly.” (Ji-Seon).

“I used to be a mechanic, then I lost my vision. In a rehabilitation center I learned various skills to live as a person with visual impairment, so I can manage everyday living anyway. Now, I am working as the director of a civil rights group for the disabled; the earnings aren’t much, but I enjoy my work because it helps individuals with visual impairment like me. As I listen to their stories, I can’t help but feel I am the lucky one among them. There are a lot of them with true hardships.” (Jeong-Ho).

#### Actively Establishing Relationships With Others as an Individual With Visual Impairment

Individuals with AVI encounter more difficulties in daily life than those with congenital visual impairment who have spent longer time without vision. From an independent gait and the use of a smart phone or a computer to activities of leisure, life after trauma for individuals with visual impairment is completely different from life before trauma. Nevertheless, the participants of this study were found to lead an active lifestyle as they established relationships with others in their changed environments. They shared the experiences they had before their impairment with other individuals who are visually impaired, while accepting the impairment as a hard fact of life. They interacted with others in the society through new behavioral patterns and strived to survive as a healthy member of the society. What they were achieving by actively establishing relationships with others included social growth, internal growth, and existential growth beyond recovery to the state before their trauma.

“There are several individuals with visual impairment among the staff at the Welfare Center, and I am the slowest among them. This is because I lost my vision only recently. So, I get a lot of help. I feel both sorry and thankful. The other staff with visual impairment express their respect and envy whenever I tell them that I was the prima donna of an opera, as I sang the best in my department at university. I try to be close to them, to be candid in conversation, to share my experiences before the trauma, and try to talk to them first and open up to them.” (Ah-Rhan).

“Working as the director of a civil rights group for the disabled, I get to meet a lot of people. There are cases that necessitate regular meetings and discussions as well as collaborative responses to certain issues, for individuals with visual impairment who visit our organization. I can never be careless about any of those cases as they all feel like my own. I feel truly happy to be able to learn about the pain or hardship of others and to help them and solve problems together. I do my best because they feel like my family.” (Jeong-Ho).

#### Re-finding Meaning of Life Through Musicing

Musicing, for the individuals with AVI in this study, was the repository of vigor that has led them to a uniquely happy and enjoyable life and the “Treasure Island” that kept replenishing itself. The participants said with a smile that they felt happy even before arriving at the place for practice, in anticipation of meeting their fellow musicers, who filled their hearts with vigor and joy. Music or musicing to them was a precious route that connected them to the memory of those days that felt like a spring day filled with sunlight and happiness and so much more but that they can never return to, as well as a space for hope and growth that has led them to accept reality as it is and to advance from it for a new leap toward a more positive lifestyle.

“Five of us are in a choir; me, my coworker at the cafe, a singing major who works at the Welfare Center, and two people with normal sight. The worker at the Welfare Center is the leader and she gives vocal lessons to me and my coworker and also coaches guitar players. Every Sunday afternoon, we get together in the bible study room of my church for practice. Sometimes, I bake bread or cookies for our team members, and everyone loves them; they said that they’d never tasted such delicious bread or cookies before.” (Yoon-Ji).

“At my church, which is for individuals with visual impairment, I sing hymns with two male vocalists with piano, guitar, and drum accompaniment, and that’s when I feel the happiest. We meet for rehearsals on Saturday evenings and play before the second and third services on Sundays. Despite all of the housekeeping work on weekdays, I memorize the lyrics of the hymns and practice singing them several times. I always await Sundays with joy.” (Ji-Seon).

“I sing the soprano part in the choir of our branch in X city, and as we are quite a well-known team, we are invited for concerts over ten times a year. It is what keeps me going, a fun and happy chance to dress up and express myself in front of others.” (Da-Hee).

“We’ve created a band, all of us with adventitious visual impairment. The keyboard player, having experience in a school band, is leading our team really well. I am the vocalist, and when all the instruments—the guitar, the keyboard, and the drums—are playing the song for me to sing, I feel so happy, as I feel like the protagonist of a story. We get together to practice and share our life stories, and we have grown really fond of each other performing in the band. Our band is the most valuable part of my life now.” (Jong-Goo).

## Discussion

This study aimed at an in-depth and contextual understanding of the key factors and processes of PTG of individuals with AVI that bring about positive changes in life. For this, the interview data of 15 individuals with AVI were analyzed, using the life history method proposed by Mandelbaum as the framework (Mandelbaum, 1973). Three domains: *dimensions*, *turnings*, and *adaptations*, were examined, and the discussion from results is as follows.

First, the *dimensions of life* of individuals with AVI were found to be the *family*, *rehabilitation center*, and *music groups*. These were the crucial parts of life for these individuals who underwent the extreme change of vision loss at midlife. These dimensions provided support for their survival and were the basis of their will to keep on living with *vigor*. For individuals who had to face unforeseen visual impairment, the family was seen as a strong source of support from the onset of impairment (Leyser and Heinze, 2001); however, the participants now feel that they should return value to the family by being successfully integrated into society. Some researchers emphasized that there was a significant negative association between family psychological resources (e.g., family emphasis on personal growth, family cohesion, family support) and stress of unique responsibilities and challenges due to increased family demands (Dyson, 1997; Sanders and Morgan, 1997; Padeliadu, 1998; Van Riper, 2000). In contrast with this, for individuals with AVI in this study, the perception of family as a source of support and the returned favor is not only interactive but also compossible, which is different from negative impacts possibly arising in families of the impaired. The rehabilitation center was a place where they could find those facing the same challenges to share the pain, consolation, and encouragement that helped them face reality and plan their future with hope. The rehabilitation center, wherein individuals with AVI could be proactive leaders of their life and become active members of the society, was the root of their pride and self-esteem that allowed rediscovering their real problems. Meanwhile, the music groups provided an opportunity for individuals with AVI to console one another through hardships; these groups served as a shelter where they could feel a sense of stability that they could not feel anywhere else, and provided a place where those facing the same challenges could embrace one another for encouragement and sharing. This finding is consistent with previous studies reporting that musicing, with an aim to improve social relationships, could help individuals with AVI to form positive relationships with others, thereby reducing their sense of isolation and promoting mutual exchange (Metell, 2015). The findings also agreed with a previous study on the function of music for social groups, which reported that music, to individuals with AVI, could make a marked contribution to promoting the exchange among group members and enabling them to acquire a sense of belonging (Park et al., 2015). Thus, music groups should be utilized actively to help individuals with AVI overcome psychosocial problems such as feelings of loss, alienation, and isolation.

Second, the *turnings of life* were found to be the *positive changes through awareness and tolerance of impairment*, *new challenges in rehabilitation training*, and *finding inner resources through music*, among which a notable positive effect was found in rehabilitation training. As these individuals could acquire various techniques through rehabilitation training to overcome the sudden challenges of impairment, they were motivated to develop a new lifestyle and achieve PTG. As in previous studies, this may highlight the importance of psychological rehabilitation in helping individuals with AVI overcome barriers to their satisfaction of life such as severe stress and feelings of anxiety, loss, isolation, and helplessness (Papadopoulos et al., 2013), while contributing to social integration. Meanwhile, individuals with AVI were found to live on with the hope that they would *get used to the impairment one day*, as they steadily increased the boundaries of everyday life with memories and accumulation of meaningful daily experiences. When compared with a previous study on musicians with visual impairment (Park, 2017), a similar result was found regarding the individual’s attitude toward the impairment among the *turnings of life*. To individuals with AVI, the impairment posed an endless problem of identifying for themselves what they had to face and solve each and every moment. In addition, musicing was found to be the core among the *turnings of life*, because the process of experiencing, enjoying, and creating music played a key role in transforming the traumatic event of visual impairment toward growth. Hence, for individuals with AVI, musicing was found to be the source of positivity that invigorated them to continue to step forward toward growth amid a challenging reality.

Third, the *adaptations of life* were found to be *accepting one’s life in a harsh social environment*, *actively establishing relationships with others as an individual with visual impairment*, and *re-finding the meaning of life through musicing*. Music and musicing were found to play a key role in enabling individuals with AVI to live on with a positive mindset, despite a harsh social environment and among those facing the same challenges. For individuals with AVI, musicing was the source of vigor amid the challenges of life, serving as a bridge to the time before their loss of vision, when life was happy, fulfilling, and wonderful, and as a place in a different dimension for survival that helped them endure through the pain brought about by the visual impairment. As such, the experience of musicing in the process of adaptation to the impairment played a central role, giving them the sense of autonomy through independent activity, satisfaction through accomplishment, and social communication or empathy, thereby ultimately leading to growth (Lee, 2015). This may be attributed to the process of creating music as a group having a significant influence on increasing not only the personal experience of accomplishment but also self-efficacy (McPherson and McCormick, 2006; Hendricks, 2016; Zelenak, 2020). Hence, music is likely to have reinvigorated individuals with AVI to live on, by letting them actively apply their remaining senses and attain a sense of immediate reward (Rostohar, 2006). In fact, the activity of creating music has been reported to help improve the self-efficacy of individuals with AVI, and the activity of listening to music was found to reduce depressive feelings (Clements-Cortes and Pascoe, 2020; Park et al., 2020). This implies that the varied experiences of musicing could help individuals with AVI to overcome the challenges of psychosocial adaptation so that they may actively communicate and empathize with others as a healthy member of the society.

Based on the discussion, the limitations of the study are as follows. First, the research was conducted on PTG, which is positive change, and therefore the positivity of the findings and their interpretations was emphasized. Individuals with AVI may have experienced pain and hardships to reach PTG, and the associated negativity they may have confronted was to some degree overlooked. In fact, the negative emotions the participants experienced in the early stages of impairment might be personal and multifaceted. Second, this study was performed on 15 individuals with AVI. Among them, 13 had an occupation and were actively engaged in economic activities. Considering the current economic and social status of individuals with AVI in South Korea, generalizing the findings in this study may be limited. Third, for this research, participants were recruited from a single facility (X community welfare center) and all of them were found to be currently participating in music activities. To accomplish the goal of this study, purposive sampling was sufficient. However, PTG can be experienced by many individuals with AVI through various aspects and characteristics, not only through the musicing thoroughly investigated in this research. In future studies, it will be necessary to conduct a more sophisticated and comprehensive study by considering their individual characteristics and impairment-related variables by using a larger sample. It is also suggested that empirical studies demonstrate the effectiveness of musicing for individuals with AVI through objective and quantitative approaches.

## Data Availability Statement

The original contributions presented in the study are included in the article/Supplementary Material, further inquiries can be directed to the corresponding author/s.

## Ethics Statement

The studies involving human participants were reviewed and approved by KU IRB 2020-0028, Kosin University Institutional Review Board. The patients/participants provided their written informed consent to participate in this study.

## Author Contributions

The author confirms being the sole contributor of this work and has approved it for publication.

## Conflict of Interest

The author declares that the research was conducted in the absence of any commercial or financial relationships that could be construed as a potential conflict of interest.
